# Intracranial Subdural Hematoma after Lumbar Spine Surgery: A Case Report

**DOI:** 10.5704/MOJ.1911.016

**Published:** 2019-11

**Authors:** AF Zakaria, M Tsuji

**Affiliations:** Department of Orthopaedic Surgery, Murayama Medical Center, Tokyo, Japan

**Keywords:** subdural hematoma, intracranial hypotension, CSF leaks, lumbar surgery

## Abstract

Intracranial subdural hematoma following lumbar surgery is a devastating but rare complication. It has been implicated due to intracranial hypotension secondary to persistent cerebrospinal fluid leakage. The resultant drop in intracranial pressure presumably causes traction and tearing of venous structures. Patients typically present with postural headaches. However, other symptoms of subdural hematoma, intracranial hypotension and cerebrospinal fluid leak must also be cautioned.

## Introduction

Spontaneous intracranial subdural hematoma (SDH) secondary to intracranial hypotension (ICH) has been reported in literature following many procedures including lumbar puncture, spinal anaesthesia and myelography^1^. However, the incidence following spinal surgery is uncommon and as low as 0.8%^2^. It is postulated that ICH is a consequence of cerebrospinal fluid (CSF) leakage. Patients typically present with severe postural headache postoperatively. This potential fatal complication must be recognised early. We describe a case of SDH developing after lumbar surgery with emphasis on clinical presentation and its immediate management.

## Case Report

**Fig. 1:**
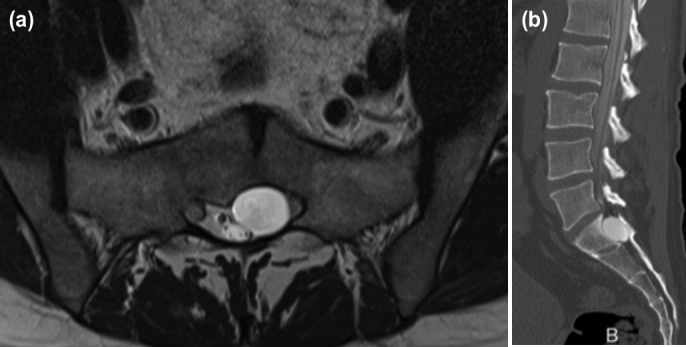
(a) MRI axial view and (b) CT sagittal view showing location of arachnoid cyst in sacral canal in close proximity with left S1 nerve root.

A 41-year-old lady complained of left sided leg pain for two months prior to presentation to hospital. Signs correlated with left S1 nerve root radiculopathy. An MRI of the spine showed left S1 nerve root arachnoid cyst measuring 3cm x 2cm x 1cm (Fig. 1). It was located adjacent to S1 foramen and at the axilla of S1 and S2 nerve root. Myelogram further denotes the relation of the cyst within the S1 nerve root when it was quickly occupied with radio-opaque contrast one-hour after injection. She was initially treated with analgesia. However, after four months of observation, she opted for surgical excision and decompression in view of persistent radicular pain.

**Fig. 2:**
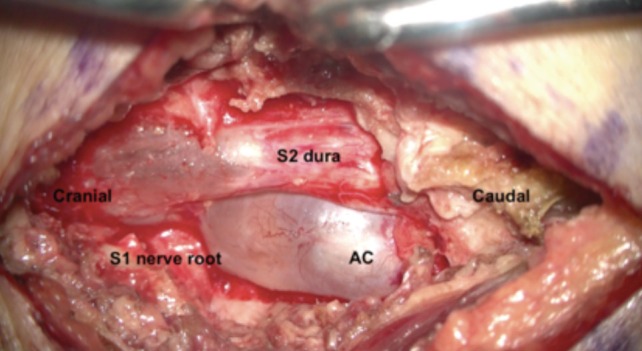
Intra-operative image of arachnoid cyst (AC) encapsulating the S1 nerve root.

A posterior midline lumbo-sacral incision was made and soft tissue retracted until sacral bone visualised. Laminectomy of S1 foramen made exposing the S1 nerve root and arachnoid cyst attaching to it (Fig. 2). Under microscopic vision, a tract at the subarachnoid space was identified as the cause of CSF collection in the cyst. Carefully the cyst was excised and the tract closed with non-absorbable sutures. This was followed by repair of the dura with non-absorbable sutures and fibrin glue. A non-vacuum lumbar drain was inserted prior to closure.

Post-operatively, patient did not sustain any neurological deficit of the left leg. However, sub-fascia lumbar drain collection was of clear fluid with persistent volume increment of 30, 70 and 300ml on post-operative day one, two and three, respectively. This was accompanied by postural headache on day two, worse upon sitting and relieved in supine position. At day three post-operatively, she experienced left sided hemiparesis.

**Fig. 3:**
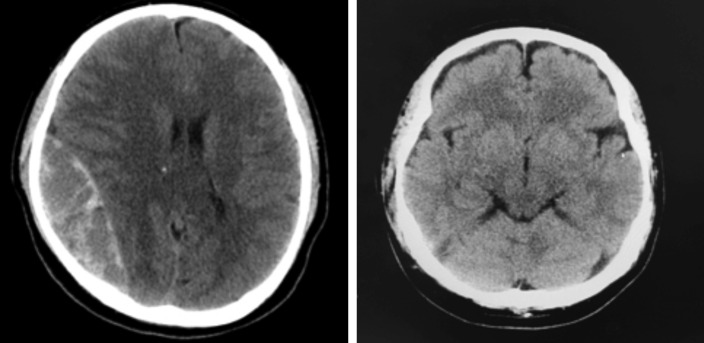
CT brain; (a) Right sided intracranial subdural hematoma at post-operative day three. (b) Resolution of hematoma after evacuation.

An urgent CT brain was done and revealed a large right-sided intracranial SDH (Fig. 3). Following that, she underwent emergency evacuation of hematoma. Lumbar drain showed reduction of CSF fluid few days after brain surgery. Further re-exploration of CSF leak was not done. Patient continued to show positive outcome with complete recovery of left sided hemiparesis.

## Discussion

Intracranial SDH is a potential fatal complication after lumbar surgery and is due to ICH. The Monro-Kellie doctrine states that intracranial pressure (ICP) is regulated by a fixed system with any change in one component will result in compensatory change in another to maintain equilibrium^3^. As the ICP drops from persistent CSF leakage, the subdural space expands. This places tension and stretching on the fragile bridging subdural veins with risks for rupture and ensuing SDH. Concurrently, caudal descent of the brain can occur predisposing to further venous tears.

Symptoms of ICH must be recognised clinically. The most characteristic is headache, occurring within 24-48 hours after surgery, worsened when standing, alleviated in supine position and tends to be bilateral in the frontal and occipital region. The etiology is likely due to resultant tension on the pain sensitive dural sinuses secondary to caudal movement of the brain^4^.

Post-operative CSF leak as the cause for ICH should be suspected clinically in presence of fluctuant subcutaneous fluid collection. Suspicion should also be raised if volumes of CSF collected in post-operative drain are between 221ml to 250ml/day^5^. A negative suction drain should not be used in cases of dural leak as it can cause rapid accumulation of CSF and lead to deterioration of ICH. Drains are normally placed sub-fascia in lumbar surgery. A passive pressure drain would be more prudent to use in cases of dural leak.

Symptoms of intracranial SDH following spinal surgery are equally important to identify. This include headache, facial palsy, cognitive dysfunction, seizure or hemiparesis among others occurring within average of three days postoperatively^2^. A total of 33% of patients became symptomatic just in the first ten hours after primary spinal surgeries with overall mortality rate of 14%^5^.

SDH is classified as acute; occurring within three days, sub acute; occurring within three days to three weeks and chronic if occurs after three weeks. According to literature, most of the patients develop within acute to sub-acute period^2^. Elderly people are likely to have delayed onset and may be attributed to increase extracerebral space secondary to brain atrophy.

Neuroimaging such as CT or MRI brain may show increase in subdural space, intracranial hemorrhage or SDH. In addition, imaging may show caudal displacement of the brain with cerebellar tonsilar herniation^1^.

Management of ICH may take a benign course with close observation, hydration, analgesia or lying supine for at least 24 hours^1^. However, its consequence resulting in SDH should be addressed promptly.

SDH are treated surgically with evacuation of hematoma especially in presence of midline shift and neurological deficit as presented in this case. In a non-severe situation, it can also be treated conservatively with close observation, fluid resuscitation, steroid, analgesics and ICP lowering agents.

Treatment strategies for ICH secondary to CSF leak after spinal surgery is limited only to case series. One literature review by Isik *et al*^2^ for 21 cases of SDH occurring after spinal surgery found that ten were treated non-operatively while remaining underwent exploration and dura repair. Though there is no concrete consensus on re-exploration or not, the decision relies on previous experience and judgement of the treating surgeon. Repair are usually done with non-absorbable suture, fibrin glue or fat/ muscle grafts. However, there is still a failure rate of 7-9%, and in this case, an unrecognised leak occurred despite good repair of the dura. Meticulous inspection intra-operatively, obtaining clear visualisation of the defect and achieving watertight closure could prevent this occurrence. A Valsalva manoeuvre, which was not done to this patient, can be used to test the suturing.

Intracranial SDH is a potential fatal complication that can occur after lumbar surgery. A thorough inspection of the dura intra-operatively is mandatory to prevent this complication. Surgeons and clinicians should be aware regarding symptoms of ICH and intracranial SDH apart from signs of CSF leak.
